# DNT Cell-based Immunotherapy: Progress and Applications

**DOI:** 10.7150/jca.39717

**Published:** 2020-03-31

**Authors:** Yingrui Li, Kang Dong, Xueke Fan, Jun Xie, Miao Wang, Songtao Fu, Qin Li

**Affiliations:** 1 Department of Biochemistry & Molecular Biology, School of Basic Medical Sciences, Shanxi Medical University, Taiyuan, 030000, China.; 2 Shanxi Pharmaceutical Group Gene Biotech co. LTD, Taiyuan, 030000, China.; 3 Department of Oncology, Beijing Friendship Hospital, Capital Medical University, Beijing, 100050, China.; 4 Department of Gastroenterology, Jincheng People's Hospital, Jincheng, 048000, China.

**Keywords:** DNTs, immunotherapy, tumor

## Abstract

Cancer immunotherapy has firmly established a dominant status in recent years. Adoptive cellular immunotherapy (ACI) is the main branch of immunotherapy. Recently, the immune effector cells of ACI, such as T cells, NK cells, and genetically engineered cells, have been used to achieve significant clinical benefits in the treatment of malignant tumors. However, the clinical applications have limitations, including toxicity, unexpectedly low efficiency, high costs and strict technical requirements. More exploration is needed to optimize ACI for cancer patients. CD3+CD4-CD8- double negative T cells (DNTs) have emerged as functional antitumor effector cells, according to the definition of adoptive immunotherapy. They constitute a kind of T cell subset that mediates nontumor antigen-restricted immunity and has important immune regulatory functions. Preclinical experiments showed that DNTs had a dual effect by killing tumor cells and inhibiting graft-versus-host disease. Notably, DNTs can be acquired from healthy donors and expanded *in vitro*; thus, allogeneic DNTs may be provided as “off-the-shelf” cellular products that can be readily available for direct clinical application. We review the progress and application of DNTs in immunotherapy. DNTs may provide some novel perspectives on cancer immunotherapy.

## Introduction

Tumor immunity refers to the treatment that activates the human immune system directly or indirectly to kill tumor cells 1, 2. In 2018, the Nobel Prize in physiology or medicine was awarded to immunologists for the discovery of treatments that suppress negative immunoregulation, which conferred mainstream status on cancer immunotherapy 3, 4. Currently, tumor immunotherapies mainly include immune checkpoint inhibitors 5-8, oncolytic viruses 9, antitumor vaccines 10, 11 and adoptive cellular immunotherapy (ACI) 12-15. ACI has been used in the clinic and has achieved great clinical benefits for malignant tumors 16, 17.

ACI amplifies and activates autologous and/or allogeneic immune functional cells *in vitro*, and then, these activated cells are infused into tumor patients to activate the immune response or kill tumor cells directly. ACI has been used in cancer patients, and this treatment includes various immune effector cells such as tumor-infiltrating lymphocytes (TILs) 18-20, natural killer cells (NK) 21, chimeric antigen receptor-modified T cells (CAR-T) 15, 22, and T cell receptor T-cells 23-25. ACI has achieved some clinical effects. For example, Zacharakis N 26 screened and expanded TILs from a patient with metastatic breast cancer and transfused these TILs back into the patient. After 42 weeks, the patient's cancer cells could no longer be detected. Maude SL 27 found that 30 children with acute lymphoblastic leukemia had a 90% remission rate after CAR-T therapy. In an ongoing phase II clinical trial (NCT03108495), the final effective rate of TILs for advanced cervical cancer was 44%; based on this result, the FDA approved TIL-based therapy LN-145 as a breakthrough treatment for advanced cervical cancer 28.

Although ACI has shown preliminary progress in cancer treatment, many obstacles must be overcome. CAR-T cells can cause severe cytotoxicity, including cytokine release syndrome and neurotoxicity. Highly specific tumor antigens are among the key elements for CAR-T therapy, and the therapeutic effect is reduced in solid tumors because of the loss of specific antigens 29. Hamieh M and coworkers 30 found that tumor cells could provide specific antigens to CAR-T cells via trogocytosis, which induces antigen loss and CAR-T cell fratricidal killing. In addition, reinfusion of these cells with modified genes increases the cost and potential risk of this treatment 31, 32. TIL therapies for malignant tumors were performed in early phase clinical trials 33, 34, and enrichment of specifically recognized TILs and complicated expansion methods made it difficult to generate therapeutically relevant T cells. Adotevi and coworkers 35 explored the effect of combining cetuximab and NK cells for patients with hepatic metastases. After reinfusion of these NK cells, the metabolic activity of most intrahepatic metastases was significantly reduced. Four months later, the patients presented with different degrees of progress and developed resistance to NK cell immunotherapy. We need more in-depth exploration to optimize ACI for cancer patients. CD3+CD4-CD8-double negative T cells (DNTs) have emerged as functional immune cells in the field of antitumor therapy because they induce high cytotoxicity in multiple tumor cells in a nonspecific manner without causing graft-versus-host disease (GVHD) 36, 37. This review covers the sources of DNTs and the research on progress made in relation to cancer immunotherapy, outlines the mechanisms by which DNTs exert immune-response killing effects and explores these outcomes as documented in experimental studies.

## The sources of DNTs

DNTs are regarded as unconventional T cells that are positive for T lymphocyte antigen receptors but negative for CD4, CD8 and CD56 surface markers. According to different T cell receptors (TCRs), DNTs are divided into two groups: αβ+DNTs and γδ+DNTs. DNTs comprise 1% to 5% of the T cells in the peripheral blood of normal humans 36, 38, 39. DNTs eliminate malignant tumors in a nontumor antigen-restricted manner, despite expressing TCR and recognizing MHC class molecules. Some studies have shown that DNTs have a strong antitumor effect 40-42. Although scientists have recently tried to describe the origin and function of DNTs, the origin and role of DNTs in immune mechanisms have not been fully revealed. DNTs have diverse patterns of expression as cell surface molecules 43 and secrete an extensive array of cytokines, and it has been suggested that they may be derived from different sources or differentiation pathways 37. The origin of DNTs also may differ among various species 44.

Human T progenitor cells develop in the thymus where they are identified as double-negative thymocytes that do not express the CD4 or CD8 T cell marker 45. Mature double-negative thymocytes gradually differentiate and pass through an immature CD4 single-positive and CD4 and CD8 double-positive phase, and eventually become a functional mature TCR-expressing CD4 or CD8 single-positive T cells, after which they leave the thymus 46-49. Some studies have suggested that DNTs may originate in the thymus to avoid negative selection, and then, DNTs are activated and expanded in the periphery 50-52. Some studies have shown that DNTs can be transformed from double-positive thymocytes 53. Crispín and coworkers 54, 55 found that human DNTs can differentiate from activated CD8+ T cells. In addition, some studies found that CD4+ T cells can transform into DNTs and that the costimulatory molecules OX40/OX40L enhance this process 56, 57. Cong M and coworkers 58 also revealed that the transformation of CD4+ T cells to DNTs is promoted by IL-2. Grishkan 59 proposed that mouse spleen CD4+ T cells stimulated *in vitro* for longer than 3 weeks can produce DNTs. However, Crispin JC 54 stimulated human CD4+ T cells for 5 days and did not produce DNTs *in vitro*.

## History of DNT-based immunotherapy

In the past, DNTs were described as regulatory T cells, and the adoptive transfer of DNTs was found to prevent allograft rejection, GVHD, and autoimmune diabetes 60, 61. Subsequently, scientists gradually discovered that DNTs had a killing effect on tumor cells. In 2003, Young KJ et al 62 injected lymphoma cells with a single class-I MHC locus mismatched spleen cells into mice and found that more than 75% of the mice survived indefinitely, and the number of DNTs increased 15-fold in the mice with the lymphoma cells compared to the number in the normal mice. The next experiment proved that DNTs induced cytotoxicity in A20 lymphoma cells *in vitro* and vivo, indicating that the DNTs had antitumor effects. Voelkl S 63 isolated a group of DNTs from the peripheral blood of melanoma patients that could specifically recognize melanoma-related antigenic peptide gp100, and could induce cytotoxicity in target cells expressing gp100 and in HLA-A2+gp100 melanoma cells. Dokouhaki et al 64 found that expanded γδ+DNTs had a killing effect on lung cancer cells *in vitro*. Merims S et al 39 found that expanded DNTs from acute myeloid leukemia (AML) patients' peripheral blood could kill both allogeneic and autologous primary leukemic blasts. This study established a method for the expansion of DNTs *in vitro* that can be used as a new immunotherapy to reduce the risk of AML recurrence in patients. Previous studies were preliminary explorations of the antitumor effects of DNTs. The findings from these studies revealed new possibilities for the use of adoptive T-cell therapy against other human cancers.

## Recent progress in DNT-based immunotherapy

Recently, many studies have explored the killing effect of DNTs on various types of cancers and determined the killing mechanism and clinical application of DNTs in cancer therapy. Xu Hong et al 65 found that DNTs inhibit the growth of pancreatic carcinoma *in vivo*. Lu Y and other studies 66 demonstrated that DNTs inhibit the growth and infiltration of human pancreatic cancer cells. Interferon-gamma (IFN-γ) factor-associated suicide (Fas)/Fas ligand (FasL) and Nrf-2 may be involved in the killing effect of DNTs. Chen B et al 67 demonstrated the feasibility and benefits of using DNTs after conventional chemotherapy. Yao J et al 68 recently reported that IL-15 enhanced the killing effect of DNTs. Fang et al 69 showed that DNT tumor infiltration increased when combined with anti-PD-1 treatment, which enhanced DNT cell-mediated antitumor effects. Andrea Ponzetta et al 70 studied the role of DNTs in the tumor microenvironment and found that neutrophils can regulate the function of αβ+DNTs and that αβ+DNTs may have an essential role in antitumor immunity. In addition, DNT research on other diseases has also shown progress. Tian Dan et al 71 adoptively transferred DNTs into an allergic asthma mouse model and found that DNTs hold potential value for treating allergic asthma. Hsu J et al 72 implemented the adoptive transfer of DNTs into a lung ischemia-reperfusion injury mouse model and demonstrated that DNTs ameliorated the lung injury. These findings provide a further understanding of the immunomodulatory functions of DNTs.

## DNT antitumor effects and mechanisms

### Ligand-receptor binding

DNTs are cytotoxic immune cells that have the inherent ability to eliminate tumor cells in a nontumor antigen-restricted manner. Fas is an important apoptotic signal receptor expressed on lymphoma cells, and it combines with FASL expressed on DNTs to induce apoptosis 17. Young et al 62 showed that DNTs killed murine A20 lymphoma cells and that the mechanism included, but was not limited to, the Fas/FasL pathway. Chen J et al 73 also showed that DNTs killed pancreatic cancer cells and that the cytotoxic effects depended on Fas/FasL. TNF-related apoptosis-inducing ligand (TRAIL) combines with its associated death receptors, including DR4 and DR5, to initiate the apoptosis pathway 74, and tumor cells exert a greater sensitivity than normal cells to TRAIL 75. Dokouhaki et al 64 found that NKG2D receptor activation promoted soluble TRAIL production of γδ+DNTs, and soluble TRAIL specifically combined with TRAIL receptors expressed on lung cancer cells to induce apoptosis 76. Yao J et al 68 showed that DNTs expressed twice as much membrane TRAIL after expanding, and the medium supernatant also detected soluble TRAIL, DNTs induced cytotoxicity in non-small cell lung cancer cells (NSCLCs) depending on the TRAIL pathway. Studies have also shown that the concentration of soluble TRAIL is related to its antitumor activity and clinical response 77. NK cells, T cells and DNTs express the active receptors NKG2D and DNAM-1. NKG2D mediates cytotoxicity by combining MHC class I polypeptide-related sequence A and B with UL16-binding protein molecules, which are highly expressed on tumor cells 62, 78. DNAM-1 induces killing by recognizing CD112 and CD155 ligands, which are highly expressed on tumor cells but negligibly expressed on their normal counterparts 68, 79-86. Lee J et al 36 demonstrated that DNTs recognize and kill acute myeloid cells mainly by activating NKG2D and DNAM-1 receptors. In leukemia cells, DNTs also upregulate antitumor activity by secreting IFN-γ, which further increases NKG2D and DNAM-1 ligand expression in leukemia cells. Xu H 65 verified that DNTs inhibit the growth of pancreatic tumors through the MICA-NKG2D pathway.

### Granzyme/perforin-mediated target cell apoptosis

Perforin, as a kind of cytotoxic molecule, forms a transmembrane tubular structure on the target cell membrane, causing cytotoxic cell death. Granzymes enter the channel formed by perforin to induce target cell apoptosis 87-89. Merims S et al 39 demonstrated that DNTs expressed higher levels of perforin than CD8+ T cells in AML patients. When blocking the secretion of perforin from DNTs, the killing effect on tumor cells decreased by 80%-97%, which showed that DNTs exerted a killing effect that depended on the perforin/Granzyme B (GZMB) pathway. Voelkl S 63 described a kind of human DNT cell clone (T4H2) that targeted melanoma cells in a perforin/GZMB-dependent manner. Lee J et al 36 showed that the inhibitory effects of DNTs on leukemia cell toxicity were largely induced by perforin/GZMB. Yao J et al 68 also showed that perforin/GZMB was involved in the process of killing NSCLCs by allogeneic DNTs.

### Cytokines

Expanded DNTs produce high levels of IFN-γ and tumor necrosis factor-α (TNF-α) *in vitro* 39, 90-92. Studies showed that the proliferation and activation of host T cells was suppressed by IFN-γ indirectly, which protected mice against GVHD 62, 93, 94. Moreover, IFN-γ was proven to have a positive therapeutic effect on human malignancies 95-97, and it could promote DNT cell-mediated killing of leukemia cells by facilitating NKG2D and DNAM-1 ligand expression 36. Furthermore, IFN-γ increased Fas and FasL protein expression to inhibit leukemia K562 cell proliferation and promote cell apoptosis 98. There has been less research on TNF-α mechanisms in relation to DNTs. Meng H et al 99 reported that TNF-α production were promoted by FasL activation in DNTs, thereby enhancing the proinflammatory effects of DNTs.

## Applications of DNTs

Cytokines activate DNT development, survival and cytotoxicity against tumor cells. DNTs also have a synergistic effect when combined with cytokines, as shown by Zhang ZX and coworkers 100, who proved that DNTs require exogenous IL-2 and IL-4 to expand and survive. In addition, IL-4 also protected DNTs from the apoptosis caused by TCR cross-stimulation 101. Liu K et al 57 showed that IL-2 stimulated the proliferation of DNTs and DNT secretion of perforin. Our previous results also showed that IL-2 administered in a coculture of DNTs and breast cancer cells enhanced the killing effect. IL-15 has a potent stimulatory effect on cytotoxic immune cells such as IL-2 102, Yao J et al 68 proposed that allogeneic DNTs effectively kill NSCLCs and inhibit the growth of xenograft tumors. Moreover, IL-15 combined with DNTs induced greater cytotoxicity in NSCLCs by increasing TRAIL production and the expression of effector molecules. Other cytokines that may affect DNTs during the acquisition of cancer immunity need to be studied. Erik Meulmeester et al 103, 104 showed that transforming growth factor-β (TGF-β) downregulated the expression of NKG2D on the surface of NK cells and inhibited the activation of NK cells by cell contact, which led us to consider whether TGF-β might also regulate the activity of DNTs in the same way. In addition, TGF-β also inhibited the activity of CD4+ and CD8+ T cells, thereby facilitating the escape of cancer cells from T cell killing and the generation of a tumor microenvironment that blocks T cell action. Therefore, DNTs may be considered in combination with TGF-β inhibitors; however, whether this combination enhances DNT cytotoxicity remains to be further studied 105, 106.

DNTs had a more substantial effect when they were combined with conventional chemotherapy 107. DNTs prevented resistance to traditional chemotherapy. Chen B 67 et al found that chemotherapy-resistant primary AML samples, after pretreatment with doxorubicin, were more sensitive to the cytotoxic effects of DNTs because of the upregulated expression of NKG2D and DNAM-1 ligands. Xu H et al 65 found that DNTs combined with gemcitabine had a better inhibitory effect on transplanted pancreatic tumors in mice. It was also reported that doxorubicin and bortezomib could upregulate the expression of NKG2D and DNAM-1 ligands on melanoma cells 108-111, promoting more effective DNT killing of tumor cells.

DNTs combined with PD-1 inhibitor therapy achieve better efficacy. Fang et al 69 found that allogeneic DNTs from healthy donors killed lung cancer cells, and the combination of a PD-1 inhibitor increased the proportion of DNT infiltration in lung cancer tissues, thus inhibiting the growth of transplanted tumors in mice. These results show that adoptive infusion of DNTs may be a promising option for chemotherapy-resistant cancer, and the combination of DNTs with PD-1 inhibitor therapy may achieve better efficacy than either treatment alone. For all these reasons, we advocate the exploration of combined therapies to unleash the full antitumor potential of DNTs.

## Advantages of DNTs

**Figure 1:**
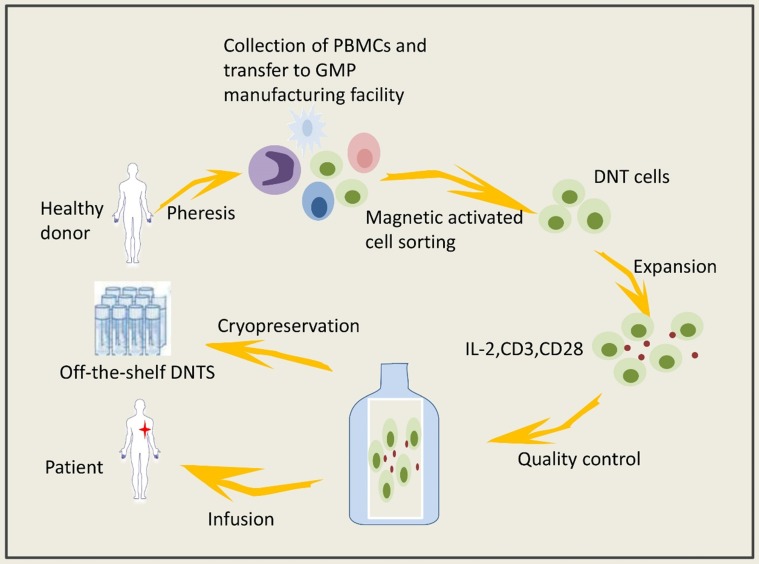
Adoptive DNT-cell based therapy involved steps.

**Figure 2:**
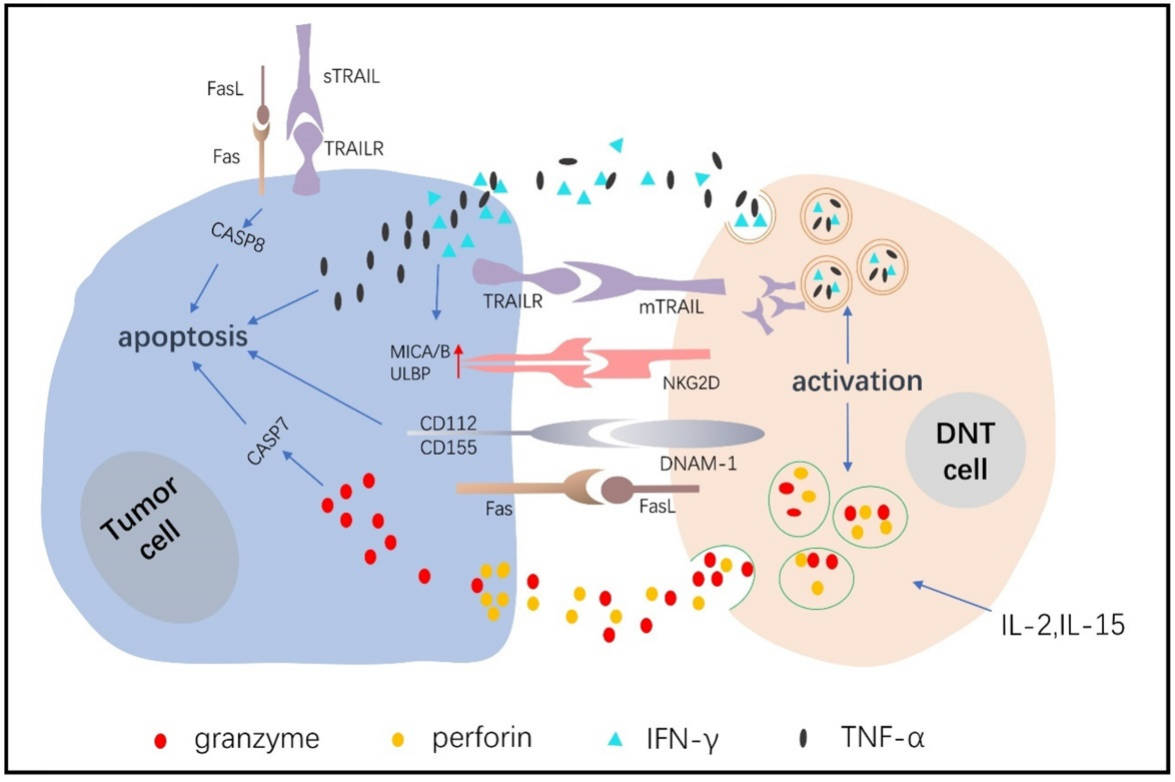
The mechanisms of DNT cells in the control of tumor cells.

DNTs can be obtained from healthy volunteers and expanded *in vitro*. Expanded DNTs cryopreserved under GMP conditions have viability and cytotoxicity that are negligibly affected. The isolation and expansion of DNTs *in vitro* could prevent the activation of various immunosuppressive mechanisms in the tumor microenvironment. Lee JB and coworkers 86 established a system to expand clinically applicable DNTs derived from healthy volunteers (Figure 1); specifically, they used cytokines to mediate the activation and proliferation of the DNTs *in vitro*, thereby preventing serious side effects caused by the application of biological agents *in vivo*. Infusion of allogeneic DNTs does not cause GVHD 112-114, and DNTs can kill cancer cells but are harmless to normal cells. Amplified DNTs can kill various tumor cells, such as lymphoma cells, melanoma cells, leukemia cells, pancreatic cancer cells and NSCLCs, via similar mechanisms (Figure 2) 36, 39, 68, 73, 115. DNTs showed superior killing against AML cell lines than did activated normal CD8+ T cells or NK cells 36. DNTs also showed significant cytotoxicity in the tumors of OCI-AML-3 and AML patients that were resistant to NK92-mediated cytotoxicity. DNTs stably present and proliferate after infusion. Young KJ et al 115 injected expanded DNTs with A20 lymphoma cells into immunodeficient mice, and the DNTs in the spleen of the mice were gradually increased and observed in the fifth month of treatment. Amplified DNTs express the effector memory T cell markers CD45RA, CD44, CD43, and CD49D, and DNTs have a longer duration during the immune response than do central memory T cells 86. In summary, allogeneic DNTs satisfy the requirements for serving as off-the-shelf ACIs without genetic modification 86, making them great clinical application prospects. Zhang and coworkers carried out a clinical trial of allogeneic DNT treatment of AML patients (NCT03027102), and the effect of the DNTs on the tumor microenvironment needs confirmation.

## Limitations of DNTs

DNTs consist of a subpopulation with the CD3+CD56-CD4-CD8- T cell phenotypes and include TCR γδ+ and TCR αβ+DNTs. DNTs in different studies show different antitumor properties due to their heterogeneity. DNTs can survive in the human body for a long time to kill tumor cells. It is beneficial for the sustained killing of tumor cells, but whether long-lived DNTs cause side effects is not completely clear. The safety of long-term application needs further verification. DNTs therapy significantly reduced lung tumor growth in patient-derived xenograft models but could not eradicate the full extent of the tumors; perhaps the lack of memory T cell formation in the immunodeficient mice was a key factor in this result. In addition, DNTs cannot continually maintain their alertness to tumor cells, and the tumor microenvironment limits the cytotoxicity of DNTs 69.

## Conclusions and Prospects

Allogeneic DNTs derived from healthy humans effectively kill tumor cells *in vitro* and *in vivo*. Compared with CAR-T therapy, DNT-based therapy does not depend on genetic modification and does not induce GVHD. DNTs can be acquired from healthy donors and used as an "off-the-shelf" ACI against cancer as promising adaptive immune cells. Summarizing previous studies, we found that most scholars found that DNTs can inhibit the growth of tumor cells. However, most of the studies were limited to cell experiments *in vitro* or animal studies. These findings cannot directly reveal the role of DNTs in the tumor microenvironment, and the role of DNTs in tumorigenesis and development is not clear. More powerful studies are needed to confirm the hypothesis that DNTs affect tumorigenesis and the microenvironment. This novel family of ACIs may provide new ideas for cancer immunotherapy and improve the long-term prognosis of patients, which is justification for the intense efforts of academic groups in this field.

## Abbreviations

ACI: adoptive cellular immunotherapy
DNTs: double negative T cells
TIL: tumor-infiltrating lymphocytes
NK: natural killer cells
CAR-T: chimeric antigen receptor - modified T cells
TCR: T cell receptors
GVHD: graft-versus-host disease
AML: acute myeloid leukemia
IFN-γ: interferon-gamma
Fas: factor associated suicide
FASL: Fas Ligand
TRAIL: TNF-related apoptosis-inducing ligand
NSCLCs: non-small cell lung cancer cells
GZMB: Granzyme B
TNF-α: tumor necrosis factor-α
TGF-β: transforming growth factor-β
